# A theoretical investigation of DNA dynamics and desolvation kinetics for zinc finger proteinZif268

**DOI:** 10.1186/1471-2164-16-S12-S5

**Published:** 2015-12-09

**Authors:** Shayoni Dutta, Yoshita Agrawal, Aditi Mishra, Jaspreet Kaur Dhanjal, Durai Sundar

**Affiliations:** 1 Department of Biochemical Engineering and Biotechnology, DBT-AIST International Laboratory for Advanced Biomedicine (DAILAB), Indian Institute of Technology (IIT) Delhi, New Delhi 110016, India

**Keywords:** ZFP-Zinc finger proteins, ns-nanosecond, desolvation energy, DNA deformation

## Abstract

**Background:**

Transcription factors, regulating the expression inventory of a cell, interact with its respective DNA subjugated by a specific recognition pattern, which if well exploited may ensure targeted genome engineering. The mostly widely studied transcription factors are zinc finger proteins that bind to its target DNA via direct and indirect recognition levels at the interaction interface. Exploiting the binding specificity and affinity of the interaction between the zinc fingers and the respective DNA can help in generating engineered zinc fingers for therapeutic applications. Experimental evidences lucidly substantiate the effect of indirect interaction like DNA deformation and desolvation kinetics, in empowering ZFPs to accomplish partial sequence specificity functioning around structural properties of DNA. Exploring the structure-function relationships of the existing zinc finger-DNA complexes at the indirect recognition level can aid in predicting the probable zinc fingers that could bind to any target DNA. Deformation energy, which defines the energy required to bend DNA from its native shape to its shape when bound to the ZFP, is an effect of indirect recognition mechanism. Water is treated as a co-reactant for unfurling the affinity studies in ZFP-DNA binding equilibria that takes into account the unavoidable change in hydration that occurs when these two solvated surfaces come into contact.

**Results:**

Aspects like desolvation and DNA deformation have been theoretically investigated based on simulations and free energy perturbation data revealing a consensus in correlating affinity and specificity as well as stability for ZFP-DNA interactions. Greater loss of water at the interaction interface of the DNA calls for binding with higher affinity, eventually distorting the DNA to a greater extent accounted by the change in major groove width and DNA tilt, stretch and rise.

**Conclusion:**

Most prediction algorithms for ZFPs do not account for water loss at the interface. The above findings may significantly affect these algorithms. Further the sequence dependent deformation in the DNA upon complexation with our prototype as well as preference of bases at the 2^nd ^and 3^rd ^position of the repeating triplet provide an absolutely new insight about the indirect interactions undergoing a change that have not been probed yet.

## Background

Genome engineering is at its inception where genome editing tools need to: help design DNA templates of choice, construction of designer proteins to manipulate DNA, implementation, testing and debugging. The current pace of development unveils the promising applications of the genome targeting tools, if large scale reengineering of genomes are carried out [1]. Evaluating literature strengthens the scope to exploit the new protein fold in ZFPs showcasing DNA binding affinity based on novel recognition principals, holding the key to engineering novel Zinc fingers for targeted genome therapy. Fingers with different triplet specificity can be engineered by mutating the key amino acid residues hence enabling specificity in DNA recognition by ensuring a large number of combinatorial possibilities. Further, linking these modules or fingers as they function independently can ascertain the recognition of longer DNA stretches[2]. Understanding how DNA molecules interact with ZFPs, critically adheres to their structure-function relationships. These relationships conspicuously deal with conformational changes in DNA and dewetting at the interaction interface of ZFP-DNA, alleviating paltry and meager aspects of affinity and specificity respectively. Characterization of binding sites is best inferred from recognition of sequence-specific contacts, mostly called direct recognition or direct readout. This mechanism highlights the "recognition code" between the key amino acid residues on the alpha-helix of ZFP and the nucleotide bases of the target DNA. Sequence dependence alone does not completely explain specificity in protein-DNA binding. Binding affinity gets afflicted by even mutating bases not in direct contact with the protein residues [3,4], implying that proteins employ modes other than direct recognition. DNA structural changes momentously affect its interactions with proteins [5]. Recognition of DNA structural properties is referred to as indirect recognition or indirect readout [6]. Governed by the binding free energy of a protein-DNA interaction, some proteins bind more strongly to certain regions of the DNA than the other regions[7]. Structural properties of DNA effecting indirect readout by proteins include flexibility, elasticity, bending and kinking, major and minor groove widths, and hydration[8-10]. The energy expended to deform DNA from its native conformation to the conformation in a protein-bound complex emphasizes on a potential recognition mechanism is the DNA deformation energy [11,12]. We have run 180 ns molecular dynamics simulations and reflected upon DNA contribution at the interaction interface based on RMSD and stability of trajectory. Further fortified by evaluating structural properties of DNA like flexibility, bending and major groove width changes across the simulation to optimize our study for DNA bending upon binding to ZFP.

**Figure 1:**
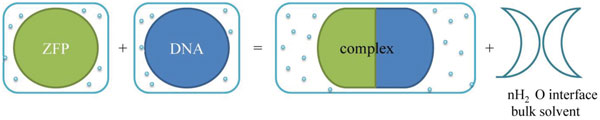
**A schematic representation of DNA-ZFP complexation and release of interfacial solvent molecules aiding strength of binding**.

Thermodynamic analyses of protein-DNA binding reveal that water released from protein DNA interfaces favors binding[13]. Structural analyses of the remaining water at the protein-DNA complex interface illustrate that bulk of these water molecules advocate binding by screening protein and DNA electrostatic repulsions between electronegative atoms/like charges. Minor fraction of the observed interfacial waters form extended hydrogen bonds between the protein and the DNA, acting as linkers compensating for the paucity of a direct hydrogen bond (Figure 1) [14].The solvent molecules equilibrate more easily and often around DNA than around the binding cavity of the protein. The degrees of freedom around the DNA are comparatively less than that of a protein-DNA complex. Hence the calculation of absolute solvation free energies is a more amenable problem than predicting binding free energies. So we have inferred from free energy perturbation data that with increasing binding affinity the desolvation energy is indicative of a stable system vouching to reach its energy minima. A study encompassing DNA-ZFP affinity and binding affected by desolvation and change in DNA conformation, without compromising on stability of respective complex as well as strong correlation with the type of template DNA is what this investigation entails.

## Computational methods

### Starting structures, models and docking studies

A sample set of eight target 9 bp DNA templates of the type 5' GNN-GNN-GNN 3', flanked by TAT-GTT-TAT, negative control in binding studies with Zif268 were used[15].These targets were docked using HADDOCK to the wild type sequence of Zif268(PDB ID: 1AAY).Ideally all 16 GNN-GNN-GNN targets have been analyzed. Since literature reveals high affinity for GC rich sequences over AT rich ones we choose only a sample set of 8 target DNA. The repeating triplet in these targets is 5' GNN 3' whose 2^nd ^(N) and 3^rd ^(N) position preference for the 4 bases have been determined. This study enables understanding of preferences between G or/and C at 2^nd ^and 3^rd ^position of the repeating triplet or A and/or T for the same positions. The sample set is given in table 1.

**Table 1:** Sample set of eight 9 bp DNA targets.

Sample target DNA sequences :5' GNN GNNGNN 3'	
GGG GGGGGG	GAA GAAGAA

GGC GGCGGC	GAT GATGAT

GCC GCCGCC	GTT GTTGTT

GCG GCGGCG	GTA GTAGTA

The 3DNA standalone software, was used to generate the 6 GNN-GNN-GNN DNA templates, which has a directory containing repeating units for each type of the 55 fibers DNA and RNA structures[16,17]. This feature allows the user to model DNA structured from just its nucleotide sequence. On choosing this mode, the user is asked to input the base sequence in the form of a data file (complete sequence) or from keyboard (only the repeating sequence). The options -a|-b|-c|-d|-z can be used for A-DNA, B-DNA, C-DNA, D-DNA and Z-DNA models respectively[18].

The HADDOCK software algorithm which is a data-driven approach to docking, utilizes distance constraints extracted from experimental data (gathered from various possible sources, such as NMR, conservation data, etc.), to reconstruct and refine the protein-DNA complex[19]. The DNA PDB files generated from 3DNA had to be converted to haddock-compatible format by removing the Chain-IDs and SegIDs. Failing to do so led the software to misinterpret the PDB files, leading to arbitrary loss of secondary structure. Restraint files were generated based on the interaction interface. Active residues, those involved in direct readout and passive residues, involving the neighboring off target sites were defined. Number of structure for rigid body docking (it0) was set from 1000 to 750 and for refinement (it1) from 200 to 100 (rate determining step). This was justified as the structure of Zif268 was extracted from its already complexed state with its consensus DNA and hence it was assumed to be close to the confirmation it would attain when docked with the new DNA. Solvated rigid body docking was not used, as the effect of solvent was determined using free energy perturbation.

### Molecular Dynamics simulation procedure

The GPU accelerated Amber molecular dynamics suite with Amber FF03force field was used to perform all atoms explicit molecular dynamics simulations (MD simulations) of protein-DNA complexes obtained upon docking http://ambermd.org/#Amber12[20-22]. The FF03 force field includes the Barcelona modification (force field pmbsc0) for nucleotide sequences mostly DNA[23] in combination with the amber all atom force field parameters for the CaDA approach using an explicit solvent model[24], was used to define parameters for docked protein-DNA complexes generated using the program HADDOCK. Since the pmbsc0 force fields biggest success is its ability to drive structures from incorrect to correct conformations, its integration with the FF03 force field will ensure conformational transitions upon minimization to get the final refined structure[25]. Further the zinc finger protein-DNA complexes containing Zn atoms were minimized using the "cationic dummy atom approach (CaDA)" which uses four identical cationic dummy atoms to mimic zinc's 4s4p^3 ^vacant orbital's which can adjust the lone-pair electrons of zinc coordinates, hereby simulating zinc's propensity for four-ligand coordination. The methods advantage lies in maintaining zinc's four ligand coordination in ZFPs in absence of harmonic restraints rigidifying the zinc-containing active sites.

Protein-DNA complex molecules were solvated with TIP3P water model [26] in a cubic periodic boundary box to generate required systems for MD simulations and systems were neutralized using appropriate number of counter ions. The distance between octahedron box wall and protein complex was set to greater than 10Å to avoid direct interaction with its own periodic image. Neutralized system was then minimized, heated up to 300 K temperature and equilibrated until the pressure and energies of systems were stabilized. Finally, equilibrated systems were used to run 30 ns long MD simulations for each. During the MD simulations, RMSD and H-bond fluctuations of DNA with protein were calculated using VMD software [27]. All simulation studies were performed on Intel Core 2 Duo CPU @ 3 GHz of HP origin with 1 GBDDR RAM and DELL T3600 workstation with 8 GB DDR RAM and NVIDIA GeForce GTX TITAN 6 GB GDDR5 Graphics Card.

### Procedure to evaluate DNA deformation upon complexation

To evaluate the DNA deformation upon binding to Zif268 for each DNA template, 3DNA software was used to identify helical parameters of the DNA template upon docking versus its conformational change upon stabilization due to MD simulation. The change in major groove width and tilt before and after complexation were evaluated using Perl scripts.

### Free Energy Perturbation method

For desolvation kinetics, the absolute or total free energy calculations that is used for computing the absolute solvation free energies by annihilating a whole solvent free molecule, was calculated using the OPLS_2005 all-atom force field with explicit solvent and were run with the default parameters in the Maestro version 9.4, interface to Desmond[28]. Using OPLS_2005-AA the intermolecular interaction energy between molecules a and b is given by the sum of interactions between the sites on the two molecules as represented by the following equation,

$$\Delta E_{ab}=\overset{on\;a}{\underset{i}{\sum }}\overset{on\;b}{\underset{j}{\sum }}\left(q_{i}q_{j}e^{2}/r_{ij}+A_{ij}/r_{ij}^{12}-C_{ij}/r_{ij}^{6}\right)$$

The non-bonded contribution to the intramolecular energy is also computed using the same expression for all pairs of sites separated by more than three bonds[29].

The docked complexes were solvated in an orthorhombic water box using a 10 Å buffer with no ions. All the simulations were run with the TIP3P water model with default parameters implemented at our in-house Multisim Facility. Since the complex contains our target DNA and protein and the protein is fixed, an absolute free energy calculation was performed. Protein in solvent and protein in vacuum was kept constant and the final energy of desolvation for the DNA was calculated. All the desolvation energies for the sample targets obtained are relative values and this method has been optimized keeping time and computation constraints in mind.

## Results and discussion

Though literature studies show high binding affinity for GC rich sequences in case of zinc finger proteins, studies uncovering the indirect interaction dynamics like stability in terms of DNA deformation and desolvation energy in this case haven't been reported so far. Our studies reveal insights about the same.

### Binding affinity determined by docking scores and respective K_D _values

Literature review based on K_D _values show that the prototype Zif268 has a K_D _value 0.4 for

5' XXXGGGXXX 3' target sequence, whereas a high K_D _value of 25 for 5' XXXGTAXXX 3' target sequence. This implies that target 5' XXXGGGXXX 3' binds to the same ZFP with higher binding affinity in comparison to the target 5' XXXGTAXXX 3' which binds with lower affinity to the same protein [30]. Docking experiments for the same targets were performed to check reliability of the docking scores. The same target 5' GGGGGGGGG 3' has a high negative docking score -150.34 revealing very high binding strength with respect to 5' GTAGTAGTA 3' which has a less negative docking score of -125.75 revealing low binding affinity (Table 2). The negative binder which ideally does not bind to a ZFP 5' TATGTTTAT 3' shows a docking score of -113.6 whereas the wild type 5' GCGTGGCGC 3' shows a docking score of -143. Thus the docking scores are reliable based on experimental K_D _data.

**Table 2:** Free energy perturbation and docking score data for our sample of 6 GNNGNNGNN target DNA bound to Zif268 protein sequence.

Target DNA sequence 5'-3'	dG Solvation (kcal/mol)	Docking Score	Protein sequence
GTTGTTGTT	-1742.44 ± 49.88	-117.87	RER RHR RER

GTAGTAGTA	-1817.5 ± 48.61	-125.75	RER RHR RER

GATGATGAT	-1905.5 ± 48.79	-124.05	RER RHR RER

GAAGAAGAA	-1952.35 ± 340.75	-114.05	RER RHR RER

GCCGCCGCC	-5150.62 ± 137.57	-129.22	RER RHR RER

GGCGGCGGC	-5156.8 ± 137.39	-131.01	RER RHR RER

GGGGGGGGG	-5411.88 ± 141.45	-150.34	RER RHR RER

GCGGCGGCG	-5460.13 ± 143.252	-134.4006	RER RHR RER

More negative the docking score stronger the binding, further the desolvation energy also enables to draw correlation that greater the binding affinity more loss of water is seen at the interface.

### Direct correlation between binding affinity and stability of complex determined by RMSD plots

**Figure 2:**
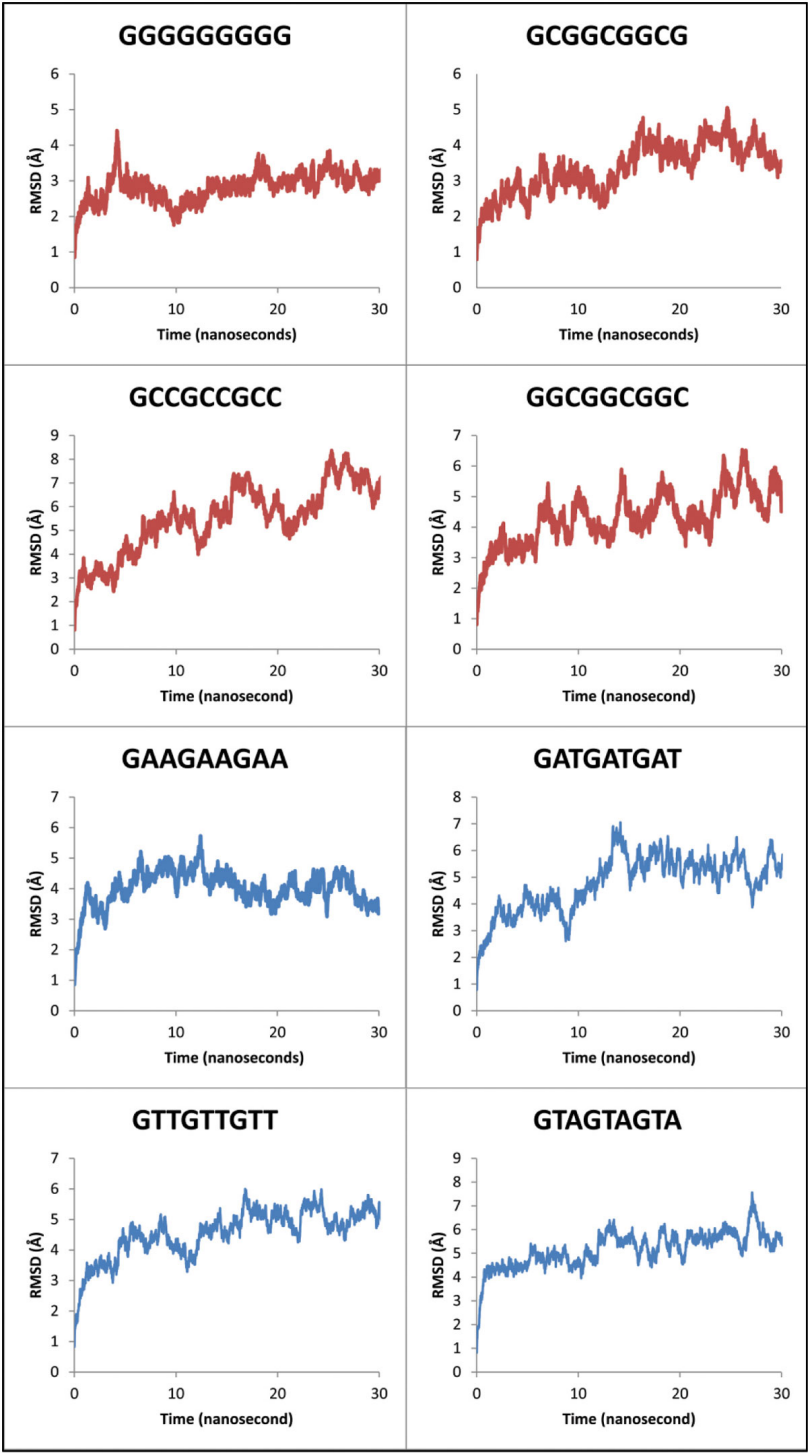
RMSD vs. time (30 ns) plot for all our target DNA sequences complexed to Zif268.

The trajectories for 180 ns simulations and respective RMSD versus total time taken for the simulation (in nanoseconds) plots were generated. This was done to ascertain the correlation between highly stable complexes possessing higher binding energies and *vice-versa*. Stability of each sequence was analyzed by the RMSD plots, where, 5' GGGGGGGGG 3' target stabilizes at 4 Å (more stability) over a time scale of 30 ns simulation trajectory as opposed to targets 5' GATGATGAT 3'and 5' GTAGTAGTA 3' stabilizing at 7 Å (less stable) over the same timescale. The target 5' GGGGGGGGG 3' has most negative docking score with very high binding strength and affinity complementing our simulation studies which exhibit relatively high stability. Similarly 5' GATGATGAT 3'and 5' GTAGTAGTA 3' have less negative docking scores with low binding affinity and lower stability based on simulation studies respectively. Hereby, direct correlation between the affinity and stability of the RMSD graphs (Figure 2) for GC rich sequences over AT rich ones was established. Variation in hydrogen bonds for each target DNA complexed with Zif268 plotted over 30 ns also substantiates our above hypothesis. Hence, strong binders have more negative docking scores, higher stability (RMSD plots) and more retention of hydrogen bonds after simulation. Similarly weak binders have less negative docking scores, lower stability and less retention of hydrogen bonds.

### Indirect interactions of Zif268 with DNA targets of the type 5' GNN-GNN-GNN 3' demonstrating the varying binding strength

#### Target DNA sequence preference by Zif268 based on hydrogen bond retention

**Figure 3:**
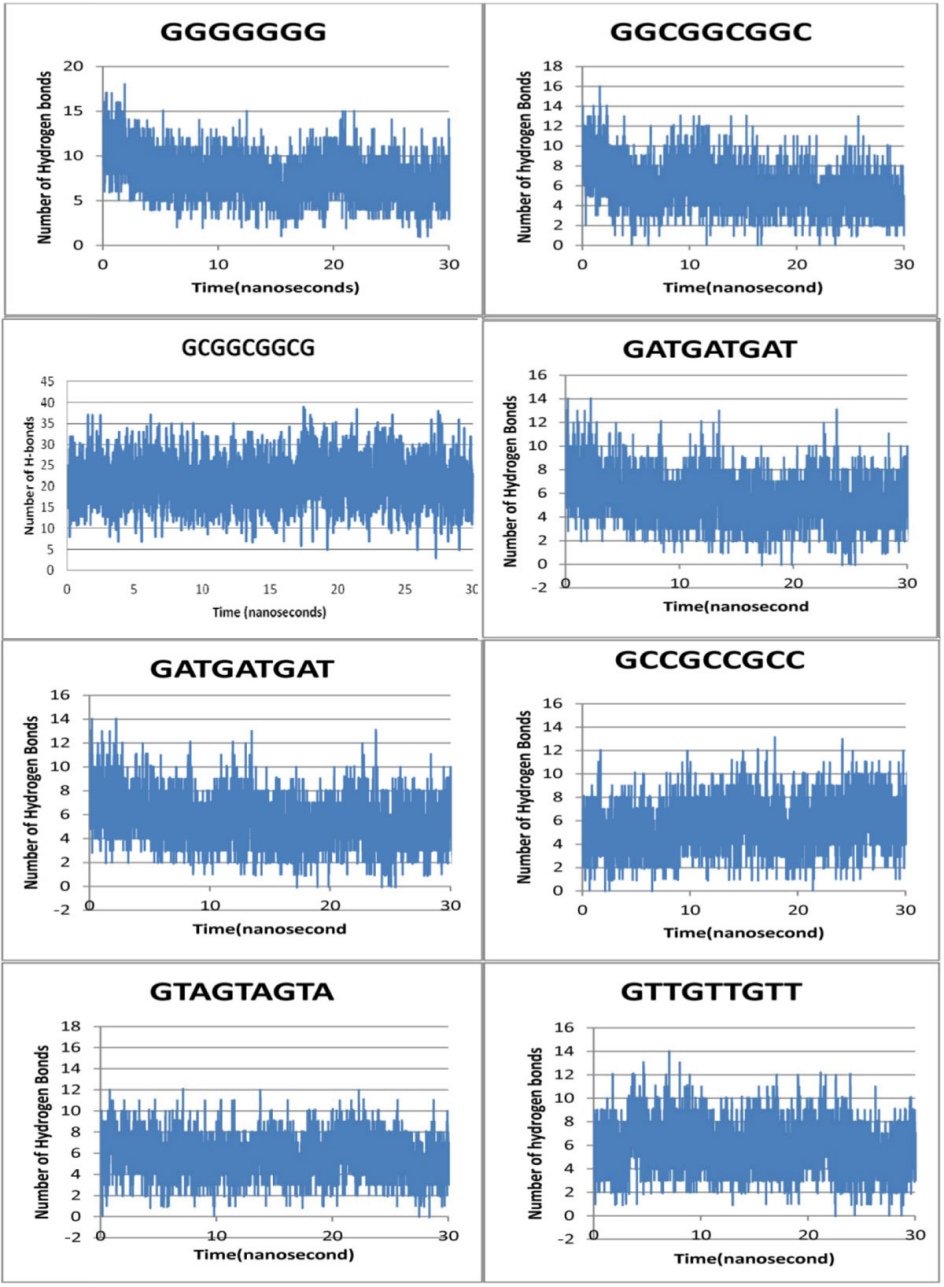
**H-bond variation over simulation trajectory our entire target DNA sequences complexed to Zif268**.

The first nucleotide of the repeating triplets in our target DNA being G, the analysis spreads to the 2^nd ^and 3^rd ^nucleotide. It was observed that, if the 2^nd ^and 3^rd ^position of the repeating triplet in our target DNA is GC rich then it is a more stable complex as compared to a AT rich one. But an interesting observation was that, the 2^nd ^and 3^rd ^position if dominated by G, shows maximum stability if not highest affinity, followed by C,A and T. Maximum numbers of H-bonds are maintained throughout the simulation trajectory for target DNA sequences rich in G at 2^nd ^and 3^rd ^base position of the repeating triplet 5' GN(2^nd^)N(3^rd^) 3' followed by G at 2^nd ^and C at 3^rd^position, then by C at 2^nd ^and 3^rd ^position of the target DNA. It emphasizes again on the heightened stability of theses complexes than for target DNA sequences rich in A at 2^nd ^and 3^rd ^position of the repeating triplet5' GN(2^nd^)N(3^rd^) 3' followed by A at 2^nd ^and T at 3^rd^position, then by T at 2^nd ^and 3^rd ^position which form lesser number of H-bond (Figure 3). Hereby, sequential preference at the 2^nd ^and 3^rd ^position of the repeating triplet by Zif268 gets a new insight.

#### Establishment of sequence-dependent DNA deformability

**Figure 4:**
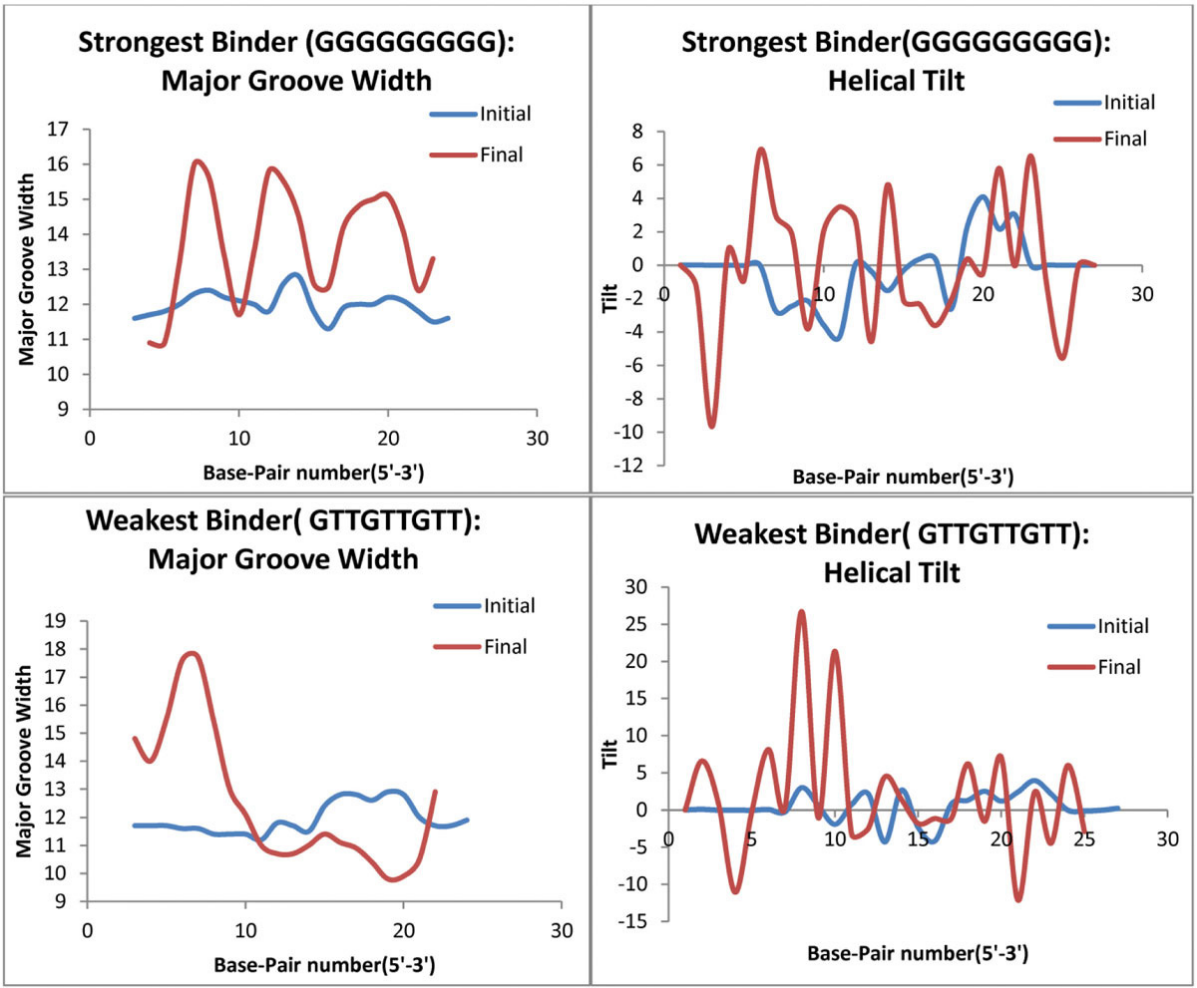
**DNA deformation as a function of Binding strength**. Parameters to evaluate conformational change in DNA like major groove width and helical tilt across the simulation trajectory for the weakest and strongest binder have been plotted.

The sequence based DNA deformation at the interface upon binding with Zif268 has been analyzed based on the change in DNA major groove width and helical tilt around the interaction interface. DNA deformation for the 2^nd ^and 3^rd^base position of the repeating triplet 5' GN (2^nd^) N (3^rd^) 3' if dominated by G (the strongest binder 5' GGGGGGGGG 3' based on negative docking scores, K_D _values and RMSD graphs) has maximum change in major groove width, followed by C and A for the same base position. If these base positions are dominated by T, least changes in the major groove width are seen (weakest binder 5' GTTGTTGTT 3') (Figure 4).Similarly the distortion in helical tilt for again the GC rich complexes is greater than the AT rich ones (Additional File 1). Fortifying the aspect that greater the deformation and conformational change in the DNA upon complexation, stronger the binding.

#### Establishment of sequence-dependent DNA desolvation

The energy required to expel water from the DNA interface upon complexation is also dependent on the target DNA sequence. The FEP values for G rich or even GC rich targets, which are the strongest binders are more negative (-5411.88 ± 141.459) revealing greater solvent loss at the interface than compared to that of the AT rich ones (-1742.44 ± 49.8897), the weakest binder. The FEP data (Table 2) shows that the 2^nd ^and 3^rd^base position of the repeating triplet 5' GN (2^nd^) N (3^rd^) 3' if dominated by G experiences greater solvent loss upon complexation followed by C and A. If these base positions are dominated by T, least solvent loss is seen at the interaction interface. Our desolvation kinetics data obtained from running free energy perturbation also corroborates the assumption in theory that greater the loss of bulk solvent at the interaction interface of ZFP-DNA complexation stronger the binding affinity and stability of the complex.

Both DNA deformation and desolvation reveal data to affirm greater deformation of DNA in case of more stable interactions followed by more negative energy needed to expel water at these interfaces.

#### Outliers

Though 5' GAAGAAGAA 3' has a less negative docking score of -114.69 and based on docking, should have been the weakest binder as compared to -117.87 of 5' GTTGTTGTT 3' but the RMSD graphs generated upon simulation show 5' GAAGAAGAA3' to be more stable than 5'GTTGTTGTT 3', even the desolvation energy follows the same preference, confirming 5' GTTGTTGTT 3' to be the weakest binder. But target 5' GCCGCCGCC 3' does not quite obey our theoretical assumptions in case of binding affinity and stability (Table 2), though it obeys indirect interactions like desolvation and DNA deformation (Additional File 1: Figure S1D). This observation might imply the strong role of indirect factors in DNA-ZFP complexation.

## Conclusion

The target DNA sequences which had strong binding affinity for Zif268 shows higher stability, greater retention of hydrogen bonds, greater deformation of its respective DNA and higher solvent loss at the interaction interface. Conversely, the weak binders show lower stability, lower retention of hydrogen bonds, lesser DNA deformation and desolvation. The binding affinity, stability, DNA deformation and desolvation are sequence dependent. These parameters favor the 2^nd ^and 3^rd^base position of the repeating triplet 5' GN (2nd) N (3rd) 3' dominated by G followed by C, A and T.

The dynamics of water molecules in the binding affinity of DNA-ZFP upon complexation has never witnessed an experimental platform and most of the tools that enable prediction of optimum ZFPs for our target DNA have overlooked it. Such a finding with the patterns unveiled can revolutionize the way we look at ZFPs for any target DNA and improve accuracy of many tools.

## Competing interests

The authors declare that they have no competing interests.

## Authors' contributions

SD and DS designed the methods and experimental setup. SD, YA and AM carried out the implementation of the various methods. SD, YA, AM, JKD and DS wrote the manuscript. All authors have read and approved the final manuscript.

## Supplementary Material

Additional file 1 **Figure S1: Interaction of Zif268 with the target DNA sequences**. DNA deformation is shown using various four parameters: major groove width, tilt, rise and stretch. Parameters like rise and stretch have been analyzed along with the major groove width and helical tilt. Stretch, rise and major groove width are translational helical parameter where stretch is evaluated at the intra-bp level and rise at the inter-bp level whereas tilt is a rotational helical parameter at the inter-bp level. **A) **5' GGG-GGG-GGG 3' strongest binder: maximum distortion in major groove width and stretch followed by tilt and rise, **B) **5' GCC-GCC-GCC 3' intermediate binder, **C) **5' GTT-GTT-GTT 3' weakest binder, **D) **5' GAA-GAA-GAA 3' weak binder. The region between 10^th ^and 20^th ^bp of the target DNA sequences. This region show maximum variation for the strong binders followed by the intermediate and weak binders for the parameters like tilt, stretch, rise and major groove width. Click here for file

## Declaration

Funding for open access charges: IIT Delhi (IRD/RP00713 to D.S.)

This article has been published as part of *BMC Genomics *Volume 16 Supplement 12, 2015: Joint 26th Genome Informatics Workshop and 14th International Conference on Bioinformatics: Genomics. The full contents of the supplement are available online at http://www.biomedcentral.com/bmcgenomics/supplements/16/S12.
