# Supplementary material for: A theoretical investigation of DNA dynamics and desolvation kinetics for zinc finger proteinZif268
Source: BMC Genomics. 2015 Dec 9;16(Suppl 12):S5. doi: 10.1186/1471-2164-16-S12-S5 (PMC4682422; doi:10.1186/1471-2164-16-S12-S5)
Supplement: Additional file 1 — Figure S1: Interaction of Zif268 with the target DNA sequences. DNA deformation is shown using various four parameters: major groove width, tilt, rise and stretch. Parameters like rise and stretch have been analyzed along with the major groove width and helical tilt. Stretch, rise and major groove width are translational helical parameter where stretch is evaluated at the intra-bp level and rise at the inter-bp level whereas tilt is a rotational helical parameter at the inter-bp level. A) 5' GGG-GGG-GGG 3' strongest binder: maximum distortion in major groove width and stretch followed by tilt and rise, B) 5' GCC-GCC-GCC 3' intermediate binder, C) 5' GTT-GTT-GTT 3' weakest binder, D) 5' GAA-GAA-GAA 3' weak binder. The region between 10th and 20th bp of the target DNA sequences. This region show maximum variation for the strong binders followed by the intermediate and weak binders for the parameters like tilt, stretch, rise and major groove width. [file 1471-2164-16-S12-S5-S1.pdf]

Figure S1: Interaction of Zif268 with the target DNA sequences. DNA deformation is shown using various four parameters: major groove width, tilt, rise and stretch. Parameters like rise and stretch have been analyzed along with the major groove width and helical tilt. Stretch, rise and major groove width are translational helical parameter where stretch is evaluated at the intra-bp level and rise at the inter-bp level whereas tilt is a rotational helical parameter at the inter-bp level.

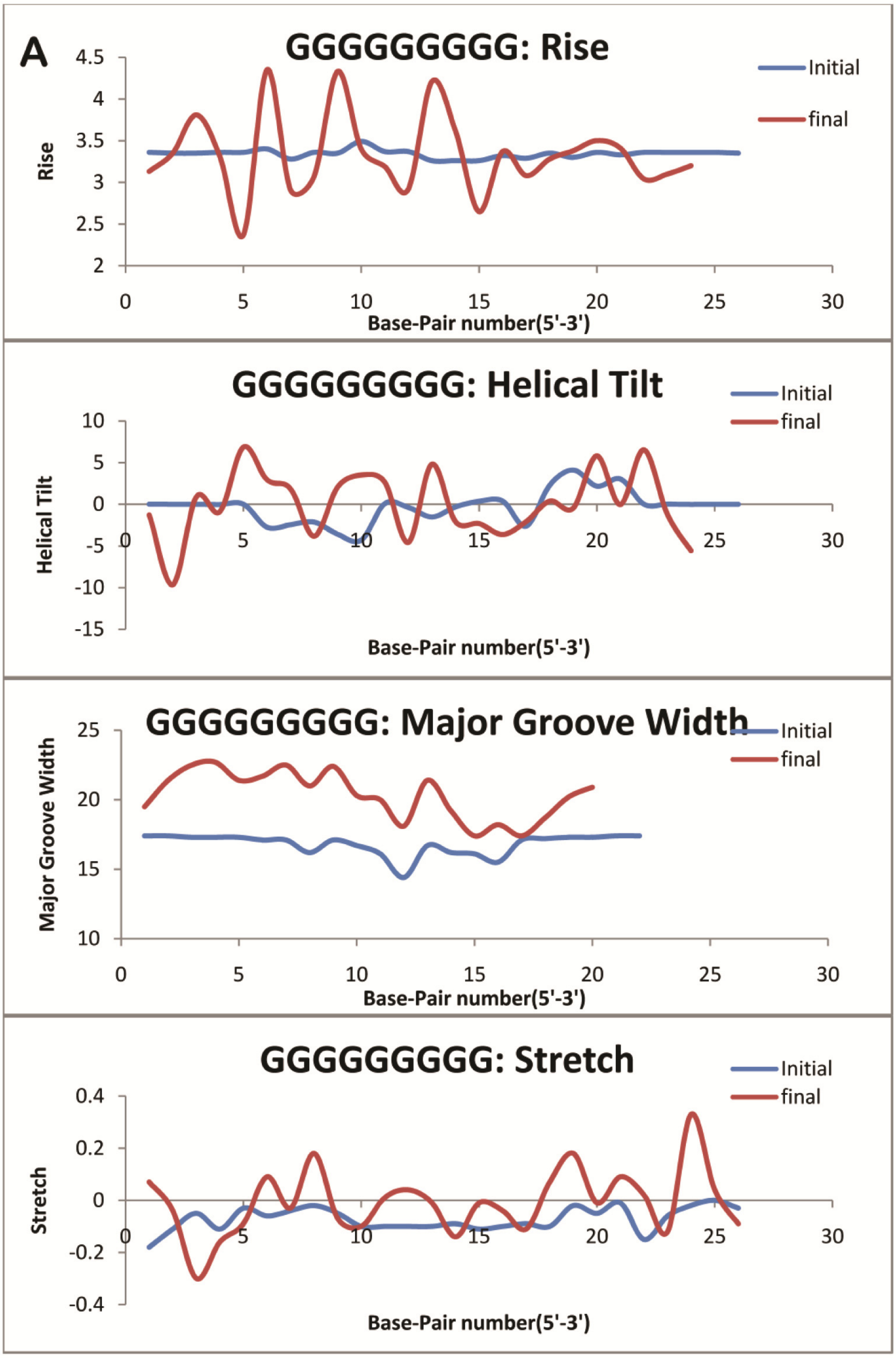

A) 5’ GGG-GGG-GGG 3’ strongest binder: maximum distortion in major groove width and stretch followed by tilt and rise

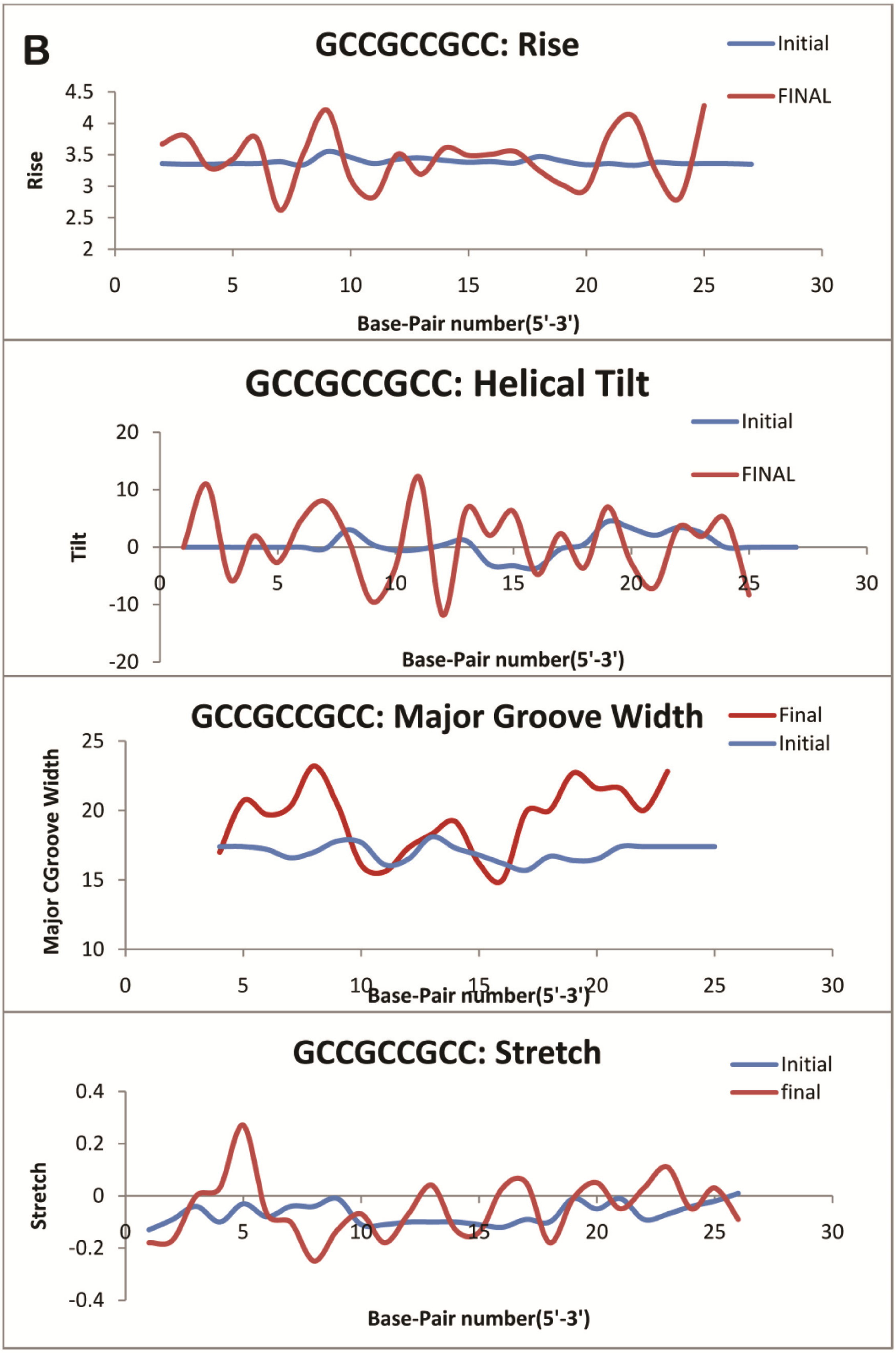

B) 5’ GCC-GCC-GCC 3’ intermediate binder

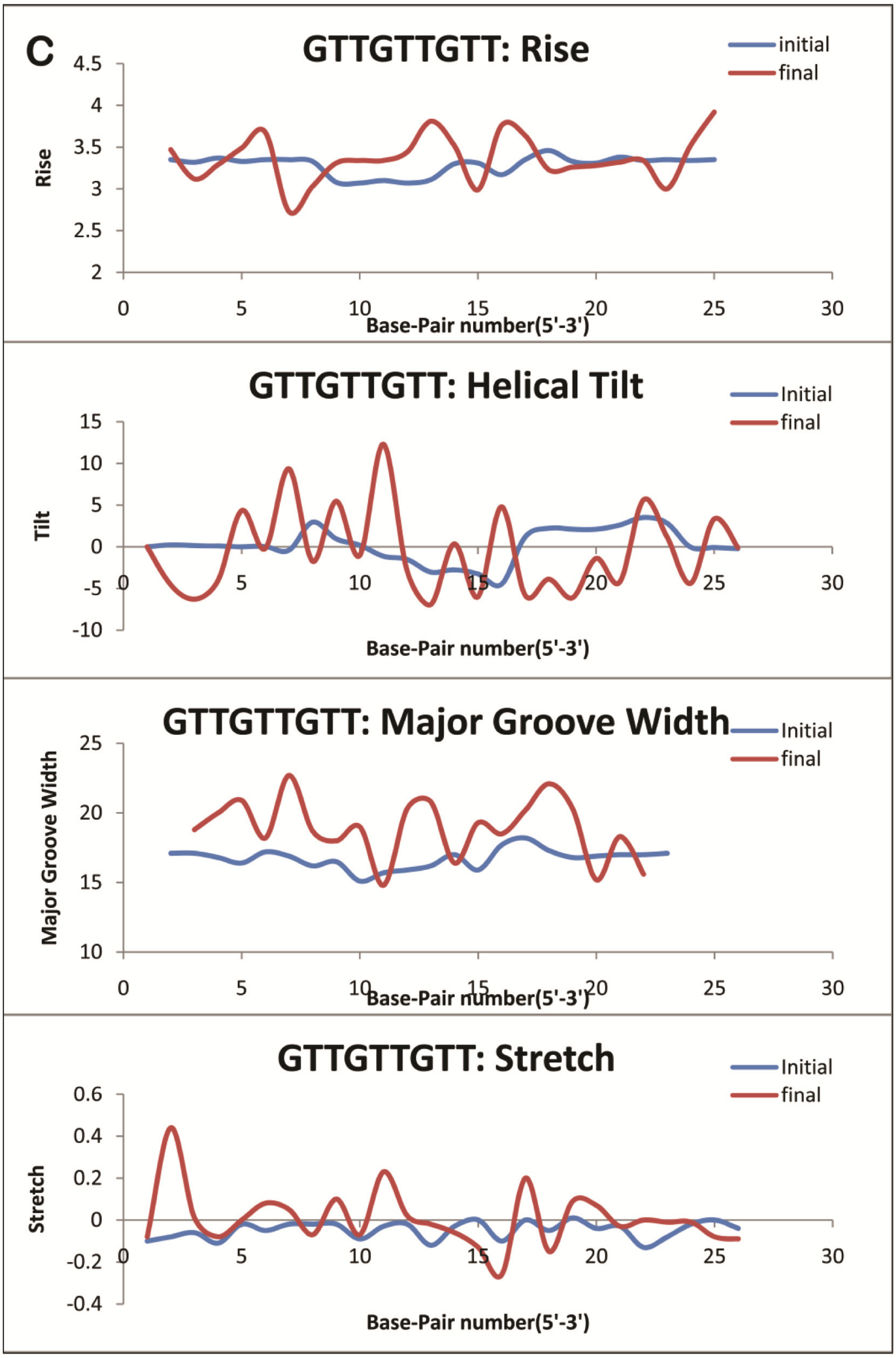

C) 5’ GTT-GTT-GTT 3’ weakest binder

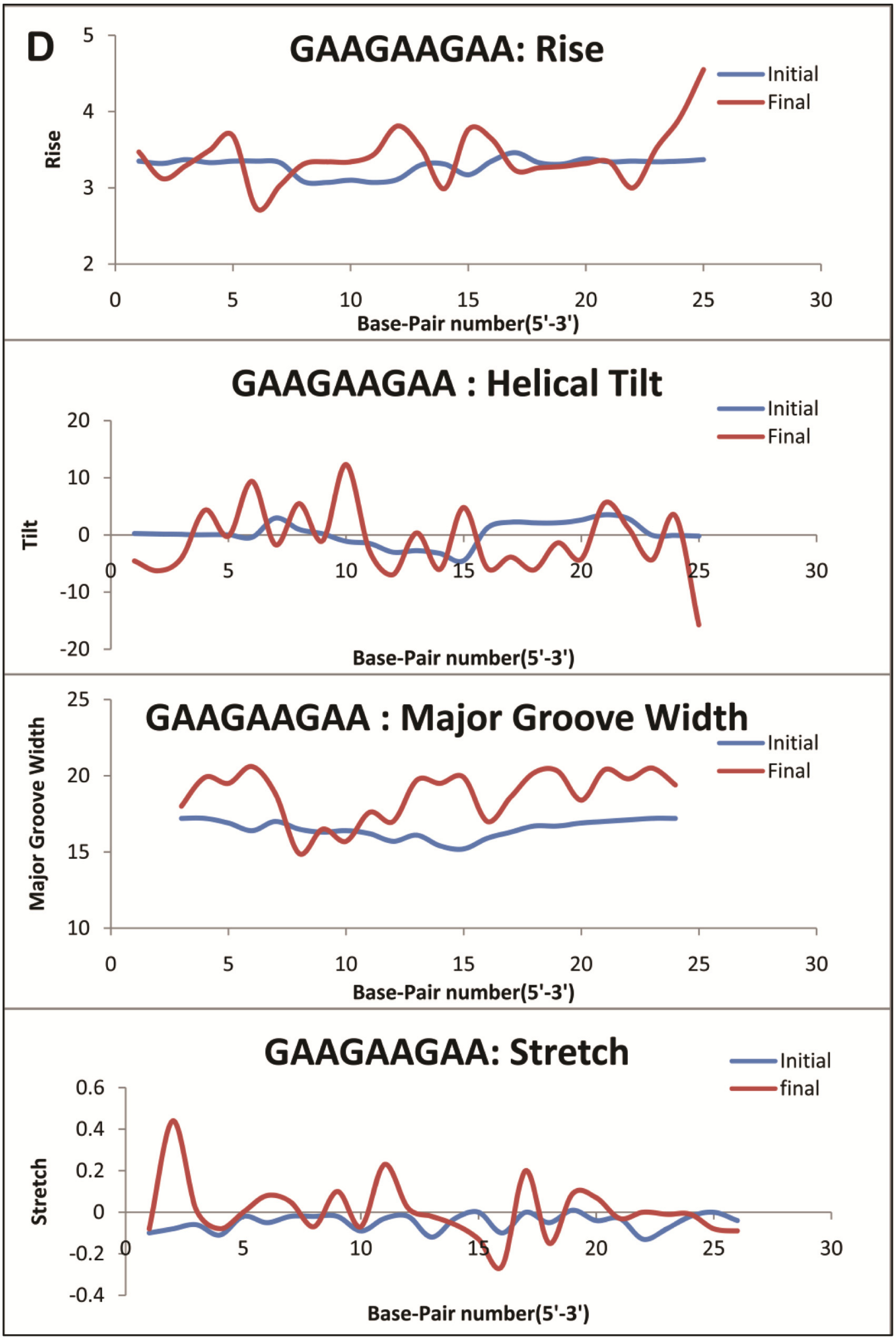

D) 5' GAA-GAA-GAA 3' weak binder.

The region between 10th and 20th bp of the target DNA sequences. This region show maximum variation for the strong binders followed by the intermediate and weak binders for the parameters like tilt, stretch, rise and major groove width.
